# The histone demethylase KDM2B regulates human primordial germ cell-like cells specification

**DOI:** 10.7150/ijbs.55873

**Published:** 2021-01-01

**Authors:** Weiyan Yuan, Zhaokai Yao, Veeramohan Veerapandian, Xinyan Yang, Xiaoman Wang, Dingyao Chen, Linzi Ma, Chaohui Li, Yi Zheng, Fang Luo, Xiao-yang Zhao

**Affiliations:** 1 State Key Laboratory of Organ Failure Research, Department of Developmental Biology, School of Basic Medical Sciences, Southern Medical University, Guangzhou, Guangdong, China; 2 Shunde Hospital of Southern Medical University, Shunde, Guangdong, China; 3 Shenzhen Hospital of Southern Medical University, Shenzhen, Guangdong, China; 4 Bioland Laboratory (Guangzhou Regenerative Medicine and Health Guangdong Laboratory), Guangzhou, China; 5 Sino-America Joint Research Center for Translational Medicine in Developmental Disabilities; 6 Department of Gynecology, Zhujiang Hospital, Southern Medical University, Guangzhou, Guangdong, China; 7 National Clinical Research Center for Kidney Disease, Guangzhou, China

**Keywords:** KDM2B, human primordial germ cell-like cells, epigenetic regulator, fertility

## Abstract

Germline specification is a fundamental step for human reproduction and this biological phenomenon possesses technical challenges to study *in vivo* as it occurs immediately after blastocyst implantation. The establishment of *in vitro* human primordial germ cell-like cells (hPGCLCs) induction system allows sophisticated characterization of human primordial germ cells (hPGCs) development. However, the underlying molecular mechanisms of hPGCLC specification are not fully elucidated. Here, we observed particularly high expression of the histone demethylase *KDM2B* in male fetal germ cells (FGCs) but not in male somatic cells. Besides, *KDM2B* shared similar expression pattern with hPGC marker genes in hPGCLCs, suggesting an important role of *KDM2B* in germ cell development. Although deletion of *KDM2B* had no significant effects on human embryonic stem cell (hESC)'s pluripotency, loss of *KDM2B* dramatically impaired hPGCLCs differentiation whereas ectopically expressed KDM2B could efficiently rescue such defect, indicating this defect was due to *KDM2B*'s loss in hPGCLC specification. Mechanistically, as revealed by the transcriptional profiling, *KDM2B* suppressed the expression of somatic genes thus inhibited somatic differentiation during hPGCLC specification. These data collectively indicate that KDM2B is an indispensable epigenetic regulator for hPGCLC specification, shedding lights on how epigenetic regulations orchestrate transcriptional events in hPGC development for future investigation.

## Introduction

Germ cells are essential to transmit genetic and epigenetic message to the next generation, defects in germline can lead to infertility and many other diseases 1, 2. In human, the primordial germ cells (PGCs) specification is a unique biological phenomenon that is established around the gastrulation stage (2nd-3rd week of the development) 3. The migrating and gonadal hPGCs in mitosis (4 to 11 weeks) exhibit their homogeneous gene expression pattern with some genes displaying developmental stage specific features. Intriguingly, the global DNA demethylation is completed from 10 to 11 weeks after gestation 4. It is discovered that the maintenance and *de novo* methylation machinery are largely undetectable during 8 to 16 weeks of development, providing direct proofs that DNA hypomethylation is retained in the germline at this stage 5. At the comparable developmental stages, FGCs from human and mouse are predominantly conserved in terms of DNA methylation and chromatin accessibility although human germline reserves unique species-specific features 6. The *in vitro* hPGCLCs specification from naïve hESCs can faithfully recapitulate *in vivo* priming and specification, which also undergoes the removal of DNA methylation 7. Although the understanding of global epigenetic reprogramming and regulation during human germline development has been studied, how specified epigenetic factor exert its role are not fully elucidated. Therefore, it is necessary to explore its correlation with transcription landscape to uncover detailed mechanisms.

Recent studies have uncovered several regulatory mechanisms during the generation of hPGCLCs which are regulated by different transcription factors (*SOX17, TFAP2C, BLIMP1, MIXL1, and EOMES*) 8, 9. During specification, from embryonic stem cells, the human PGCs (hPGCs) display hallmarks of epigenetic reprogramming, such as genome-wide DNA demethylation, imprint erasure, X chromosome reactivation as well as rearrangement of chromatin modifications 10. It is not fully understood that how these epigenetic regulation networks regulate hPGCs specification. The histone lysine demethylase KDM2B (also known as JHDM1B, FBXL10) contains a histone lysine demethylase catalytic domain, JmjC, which catalyzes the demethylation of H3K4me3 and H3K36me2 11-15. Besides, KDM2B protects the polycomb-occupied promoters against ectopic *de novo* methylation 16. Furthermore, KDM2B is identified as one subunit of non-canonical PRC1.1 complex, which can be recruited to CpG islands 15, 17, 18. Of note, the CpG sites are highly enriched in the promoters of most development-related genes, thus the epigenetic modification at this position usually imply special biological significance.

Previous studies have shown that KDM2B is involved in maintenance of pluripotency in mouse embryonic stem cells (mESCs) status whereas it's dispensable for human embryonic stem cells (hESC)'s 17, 19, 20. For instance, KDM2B deficiency had no significant effect on hESC's pluripotency and SOX17^+^ endoderm precursors generation, but PAX6^+^ neuroectoderm formation were exclusively abolished 20. Another study reported that *Kdm2b^∆CxxC/∆CxxC^* (deletion of the CxxC domain) mice were embryonic lethal 21. Moreover, it was observed that the* Kdm2b*'s expression in testis was higher than any other tissues or organs in adult mice 22. Mice carried *Kdm2b*^∆J/∆J^ mutation (deletion of the JmjC domain) had only half sperms as compared to wild-type (WT) 22. Testes from 7-month-old *Kdm2b*^∆J/∆J^ mice contained a much higher ratio of seminiferous tubules exhibiting spermatogenesis' disorders which were correlated with drastic alteration of H3K4me3 distribution in testicular germ cells, indicating that KDM2B plays a key role in sustaining spermatogenesis via regulating H3K4me3 status in testicular germ cells 11.

In this study, we explored the potential role of KDM2B throughout hPGCLCs' differentiation by generating *KDM2B* knockout (KO) cell line using CRISPR/ Cas9 system. Using time-course RNA-Seq, we comparatively investigated the transcriptional variations upon *KDM2B* deletion in hPGCLCs with the WT counterpart. Finally, with functional rescue experiment by induced overexpression of KDM2B, we demonstrate the importance of KDM2B associated epigenetic network during hPGCLC's development.

## Methods

### Culture of Human Embryonic Stem Cells

The human embryonic stem cell lines (hESCs, Fy-hES-3) were cultured in feeder-free condition (mTeSR1, Stemcell Technology) medium on Matrigel (354277, Corning). Cells were passaged every 4 to 5 days using EDTA. 10 μM ROCK inhibitor (Y-27632, 1254, Tocris bioscience) was added to the media for 24 hrs after each passage.

### Induction of 4i hESCs and hPGCLCs

The 4i hESCs were cultured on Mitomycin-treated mouse embryonic fibroblasts (MEFs) in Knockout DMEM supplemented with 20% knockout serum replacement (KSR), 0.1 mM nonessential amino acids (NEAA), 2 mM L-glutamine, 0.1 mM β-mercaptoethanol, 20 ng/ml human LIF (7734-LF-500, R&D Systems), 8 ng/ml bFGF (233-FB-001, R&D Systems), 1 ng/ml TGF-β1 (100-21, Peprotech), 3 µM CHIR99021 (4423, Tocris Bioscience), 1 µM PD0325901 (4192, Tocris Bioscience), 5 µM SB203580 (1202, Tocris Bioscience), and 5 µM SP600125 (1496, Tocris Bioscience). 4i hESCs were induced for 3 to 5 days, and 10 μM ROCK inhibitor was added for 24 hrs after the induction. 4i hESCs were dissociated with TrypLE Express and plated into ultra-low cell attachment U-bottom 96-well plates (7007, Corning) at a density of 4,000-5,000 cells/well in GK15 medium (GMEM with 15% KSR, 0.1 mM NEAA, 2 mM L-glutamine, 1 mM sodium pyruvate, and 0.1 mM β-mercaptoethanol) containing 300 ng/ml of BMP4 (314-BP-01M, R&D Systems), 100 ng/ml SCF (255-SC-001, R&D Systems), 100 ng/ml LIF (7734-LF-500, R&D Systems), 50 ng/ml EGF (236-EG-01M, R&D Systems) and 10 μM ROCK inhibitor.

### Fluorescent Activated Cell Sorting (FACS)

The cell aggregates were incubated in 0.25% Trypsin-EDTA (15400-054, GIBCO) at 37°C for 15 min. The dissociates were quenched by FBS, followed by pipetting to generate a single-cell suspension. To analyze or sort hPGCLCs with cell surface markers, samples were stained with APC-conjugated anti-human CD326 (EpCAM) antibody (324208, Biolegend) and BV421-conjugated anti-human/mouse CD49f (INTEGRINa6) antibody (313624, Biolegend) at 4°C for 15 min. The samples were loaded on a MoFlo XDP (Beckman Coulter).

### Generation of knockout cell lines

In order to knock out *KDM2B* gene, guide RNAs (gRNA) targeting exon 7 of *KDM2B* were designed and cloned into pX330 vector. 10 μg pX330 constructs containing gRNA were electroporated into Fy-hES-3 cells using Neon^TM^ transfection system (MPK10096, Thermofisher). Two days later, the top 1% GFP positive cells were sorted by FACS and picked manually into matrigel-coated 96-well-plate at density of single cell per well and cultured in mTeSR1 medium containing 10 μM ROCK inhibitor. After 3 days, the medium was changed to fresh mTeSR1 medium and one week later until passage. Between 12 to 15 days, the surviving clones were passaged into 24-well plates and half of the cells were harvested for genotyping. The targeted deletion of exon 7 loci in *KDM2B* was assessed by Sanger sequencing. The losses of targeted deletion in *KDM2B* KO cell lines were further validated by Western blot and immunostaining. Detailed oligonucleotides used are listed in Table S1.

### Lentivirus Preparation and Transduction

Human *KDM2B* was amplified from cDNA and cloned into a doxycyclin-inducible lentiviral vector to generate TetOn-*KDM2B*-3xflag-EGFP plasmid. Lentivirus was prepared by co-transfection of TetOn-*KDM2B*-3xflag-EGFP plasmid with pMD2.G and psPAX into 293T cells, and collected after 48 hrs of transfection. Viral supernatant was filtered through a 0.45-µm membrane and concentrated by a spin column (UFC901096, Millipore) before being applied to *KDM2B* KO hESCs. Approximately 10 thousand hESCs were added to each well of 24 well plates and each well was infected with lentivirus. After 24 hrs, doxycyclin was added to induce *KDM2B* expression. At 48 hrs after induction, then the cells were sorted for EGFP expression and then seeded onto Matrigel-coated 96-well plates at a density of a single cell per well.

### Induction of Teratoma in Mice and Histology

Approximately 2 million WT or *KDM2B* KO hESCs were injected under the skin of anesthetized severe combined immunodeficient (SCID) mice. After 8 weeks, mice were sacrificed and tumors were excised. For histology, teratoma were fixed in 4% paraformaldehyde, embedded in paraffin and sliced into 5 µM sections. Histological slides were stained with hematoxylin and eosin and analyzed.

### Karyotype Analysis

To obtain the metaphase from hESCs were harvested when the cells reached 60%-80% confluency in 6-cm dish. Cells were incubated with the culture medium containing 250 ng/ml of demecolcine (D1925, Sigma Aldrich) for 2 hrs at 37℃ in a 5% CO_2_ incubator in order to attain the metaphase arrest. After dissociated of cell aggregate by TrypLE Express, cells were collected by centrifuged and 2 ml new hypotonic solution was added to the cell pellet. Cells were then fixed with Carnoy's solution (3:1 mixture of methanol and acetic acid), dropped onto pre-chilled glass slide. The metaphase spreads of the cells were stained with Giemsa for 20 min. Karyotype images were obtained with a fluorescence microscope from Carl zeiss (Axio Imager.A2, Zeiss). The number of chromosomes was counted manually. Chromosomes from at least 20 random metaphase-arrested cells were counted per sample.

### Real-time Quantitative PCR

Total RNA was extracted by Trizol™ (15596026, Invitrogen) according to the manufacturer's recommendations. Reverse transcription reactions were performed using HiScript QRT SuperMix for qPCR (R123-01, Vazyme). Real-time quantitative PCR was performed using 2X PCR master mix (A301-10, GenStar) on LightCycler96 TM system (Roche). The expression level of genes of interest was normalized to the expression of housekeeping gene GAPDH according to 2^-ΔΔCT^ formula. The primer sequences used in this study are listed in Supplementary Table S2. Error bars are mean ± SD from two or three independent experiments.

### Immunofluorescence

The cell aggregates were fixed with 4% paraformaldehyde for 3-6 hrs at 4°C. They were washed three times with PBS containing 0.2% Tween-20 (PBST), and replaced with serial concentrations (10% and 30%) of sucrose in PBS overnight at 4°C. The samples were embedded in the OCT compound (Tissue-Tek), frozen, and cryo-sectioned at a thickness of 10 µm. The sections were placed on a glass slide. They were washed with PBS three times, then incubated in blocking solution (PBST containing 5% bovine serum albumin) for 1 hr at room temperature, followed by incubation with the primary antibodies in blocking solution overnight at 4˚C. The sections were washed three times with PBS and incubated with the secondary antibodies and 10 μg/ml Hoechst 33342 in blocking solution for 1 hr at room temperature in darkness. They were then washed three times in PBS, and mounted in mounting medium (S2100, Solarbio).

For immunofluorescence of hESCs, the clones were cultured on circular slides and fixed with 4% paraformaldehyde in PBS for 30 min, washed three times with PBST. Then the slides were incubated in PBST containing 5% bovine serum albumin for 1 hrs at room temperature followed by incubation with primary antibodies in blocking solution overnight at 4˚C. The slides were washed three times with PBS and incubated with the secondary antibodies and 10 μg/ml Hoechst 33342 in blocking solution for 1 hr at room temperature in darkness. They were then washed three times in PBS, and mounted in mounting medium (S2100, Solarbio). Images were taken by confocal laser scanning microscope (Carl Zeiss LSM 880). All antibodies used in this study are listed in Table S3.

### Western Blot

The cells were lysated in RIPA Lysis and run on 10% SDS-polyacrylamide gel and transferred to PVDF membranes (RPN303F, GE). The primary antibodies are listed in Supplementary Table S3. The secondary antibodies used include anti-rabbit HRP (ZSJB-BIO, zb2301) and anti-mouse HRP (ZSJB-BIO, zb2305). The ECL Western Blotting Substrate Kit (YEASON, 36208ES60) was used on the membrane before exposure. The immunoblots were quantified by measuring the relative gray-scale intensity of the protein bands with ImageJ software (http://imagej.nih.gov/ij/).

### RNA Isolation and Library Generation

Total RNA was isolated using TRIzol™ Reagent (15596026, Invitrogen) and cDNA libraries were generated using NEBNext Ultra™ II Directional RNA Library Prep Kit for Illumina (E7760L, NEB). The Next Generation Sequencing (NGS) libraries were prepared using KAPA Hyper Prep Kit (KK8505, KAPABIOSYSTEMS). All the NGS libraries were quantified using Equalbit dsDNA HS Assay Kit (EQ111-01, Vazyme) using Qubit™ 4 Fluorometer (Q33226, Invitrogen). The NGS libraries were subjected to paired-end (PE) 150 bp sequencing in Illumina Hiseq XTEN platform at Novogene.

### RNA-Seq Data Analysis

RSEM integrated bowtie2 23 was used to build reference transcriptome from hg38 reference genome using GFT-ensemble version 95. Then the 150 bp RNA-Seq paired end reads were aligned to reference transcriptome hg38 and each gene read counts were calculated using RSEM. The library is then subjected to normalization for GC content using EDAseq 24. The low expressed gene were discarded by cutoff (row mean counts >= 50). Differential expression was assessed via DESeq2 25. The detail differentially expressed gene list has been provided as Supplemental document file. The gene intersections were performed using R-package (VennDiagram). Gene ontology (GO) analysis was done using METASCAPE (www.metascape.org). The Gene set enrichment analysis (GSEA) was performed using version 4.0.3.

### Statistical Analysis

Statistical tests in this study include One-way ANOVA, Student's t-test and Wilcoxon-rank-sum test. P-values <0.05 is accepted as statistical different.

### Data Availability

RNA sequencing data of this work have been deposited to NCBI GEO under accession code GSE160287.

## Results

### KDM2B is highly expressed in human fetal germ cells

**Figure 1:**
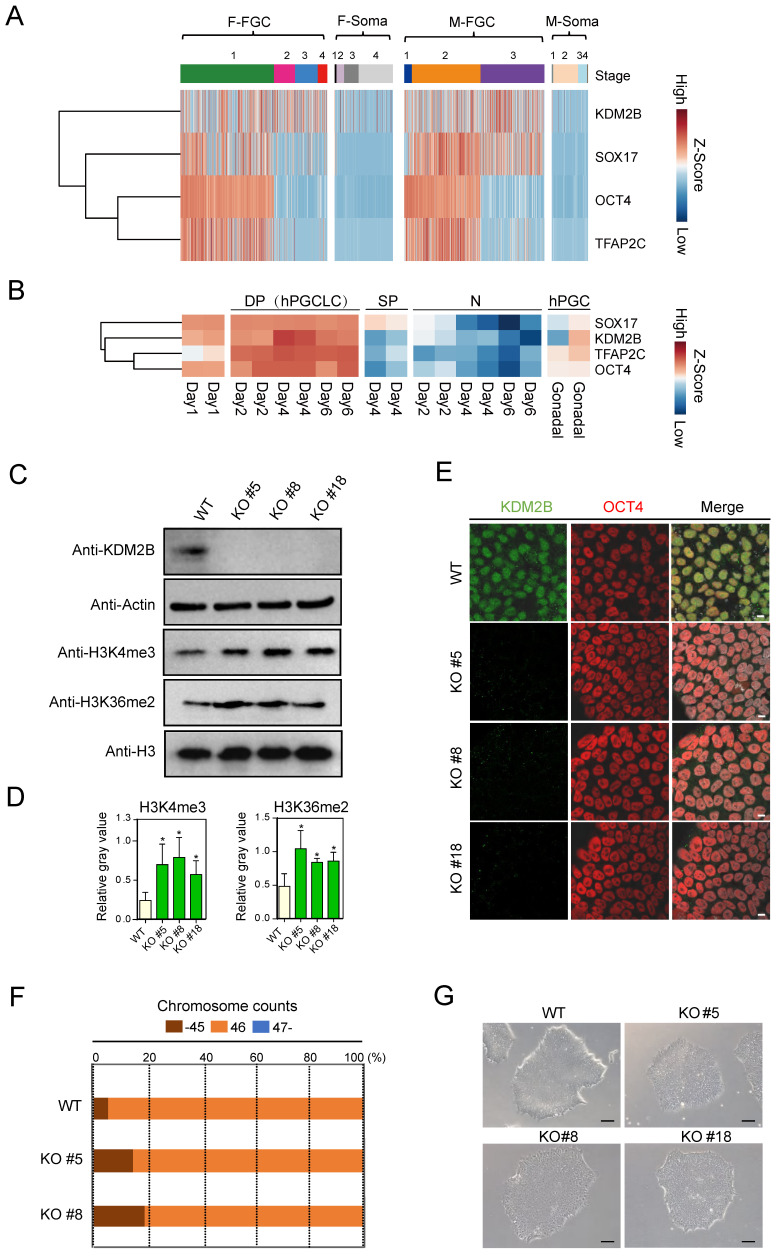
** KDM2B is highly expressed in human FGCs and hPGCLCs.** (A) Heatmap analysis of KDM2B and PGC marker genes in FGCs and somatic cells. F-FGC means female FGC (F-FGC-1: 5-10 week, F-FGC-2: 11-13 week, F-FGC-3: 14-17 week, F-FGC-4: 18-26 week), F-Soma refers to female somatic cells (F-Soma-1: endothelial cell, F-Soma-2: early granulosa cell, F-Soma-3: mural granulosa cell, F-Soma-4: late granulosa cell). M-FGC represents male FGC (M-FGC-1: 4 week, M-FGC-2: 5-8 week, M-FGC-3: 9-25 week), M-Soma refers to male somatic cells (M-Soma-1: endothelial cell, M-Soma-2: sertoli cell, M-Soma-3: leydig precursor cell, M-Soma-4: differentiated leydig cell). (B) Heatmap analysis of *KDM2B* and hPGCLC specific genes during hPGCLC generation. EpCAM+/INTEGRINα6+ double positive cells are represented as DP, EpCAM or INTEGRINα6 single positive cells are represented as SP, EpCAM-/INTEGRINα6- cells are represented as N. The color key from blue to red indicates low to high expression levels, respectively. (C) Western blot analyses show the abolishment of KDM2B protein expression in all three *KDM2B* KO hESCs (KO #5, KO #8, KO #18) and upregulated levels of H3K4me3 and H3K36me2. Actin and histone H3 were served as loading controls. In all panels, one representative experiment is shown out of the three replicate experiments. (D) Immunofluorescence analysis of KDM2B expression in WT and *KDM2B* KO hESCs (KO #5, KO #8, KO #18). Scale bars, 10 µm. In all panels, one representative experiment is shown out of the three replicate experiments. (E) The relative gray values of H3K4me3 or H3K36me2 to H3 in (C) was assessed using ImageJ software. Error bars indicate mean ± SD, n = 3 in (C). ∗P < 0.05. (F) Karyotypes represented by the percentages of the indicated chromosome numbers in WT or *KDM2B* KO #5, #8 hESCs. The color-coding is as indicated. A phase-contrast image of WT and *KDM2B* KO #5, #8 and #18 hESCs. Scale bars, 250 µm.

By re-analyzing previously published scRNA-Seq database (GSE86146) 26, we found *KDM2B* was highly expressed in both female and male FGCs, but not in somatic cells, which share similar expression pattern with PGC marker genes such as *SOX17, OCT4* and *TFAP2C* 27 (Figure 1A). Furthermore, we examined whether *KDM2B* exhibits similar expression pattern with hPGCLC marker genes in another dataset (GSE143345). Using well defined sorting strategies 8, we performed RNA-Seq with EpCAM and INTEGRINα6 double positive hPGCLCs, EpCAM or INTEGRINᾳ6 single positive as well as double negative cell populations. Compared to other cell types, *KDM2B* was only actively transcribed in hPGCLCs (Figure 1B), suggesting an important role for *KDM2B* in germ cell development.

### Generation of *KDM2B* Knockout hESCs

To investigate the molecular basis of KDM2B in primed hESCs, we generated targeted deletions of KDM2B in hESCs using CRISPR/ Cas9 in the karyotypically normal hESC lines 28. Guide RNAs (gRNAs, Table S1) targeted exon 7 (N-terminal portion of the JmjC domain) of human *KDM2B* were designed and subsequently cloned into plasmids which could co-express gRNAs and Cas9. Next, electroporation was performed and individual hESC clones were picked and genotyped.

A total of 17 clones were analyzed, among them, three homozytic *KDM2B* knockout clones (namely *KDM2B* KO #5, #8 and #18 hereafter) were selected for further analysis (Figure S1A). To identify and confirm the precise mutation, we performed PCR followed by Sanger sequencing of individual alleles with these *KDM2B* KO hESC lines (Figure S1B, C). The abolishment of KDM2B protein expression in all three *KDM2B* KO hESCs was confirmed by both immunoblotting (Figure 1C) and immunofluorescence assays (Figure 1D). Interestingly, deletion of *KDM2B* induced upregulated levels of H3K4me3 and H3K36me2 in hESC (Figure 1C, E), which was consistent with previous findings in mESC 13, 29, 30. The karyotype assay and microscopic evaluation with *KDM2B* KO hESCs after 15 passages suggested that *KDM2B* deficiency didn't impair chromosome stability and cell morphology (Figure 1F, G). Furthermore, the expression of classical pluripotency markers including OCT4, NANOG and SOX2 in *KDM2B* KO hESCs were comparable to WT controls (Figure S2A, B), and *KDM2B* KO hESCs were capable to form teratomas with three somatic lineages when transplanted into immunocompromised mice (Figure S2C). These data indicated that KDM2B had no significant impact on the cell pluripotency in human ESCs, which is consistent with the previous report 19.

### Loss of KDM2B in hESCs impairs the hPGCLC generation

**Figure 2:**
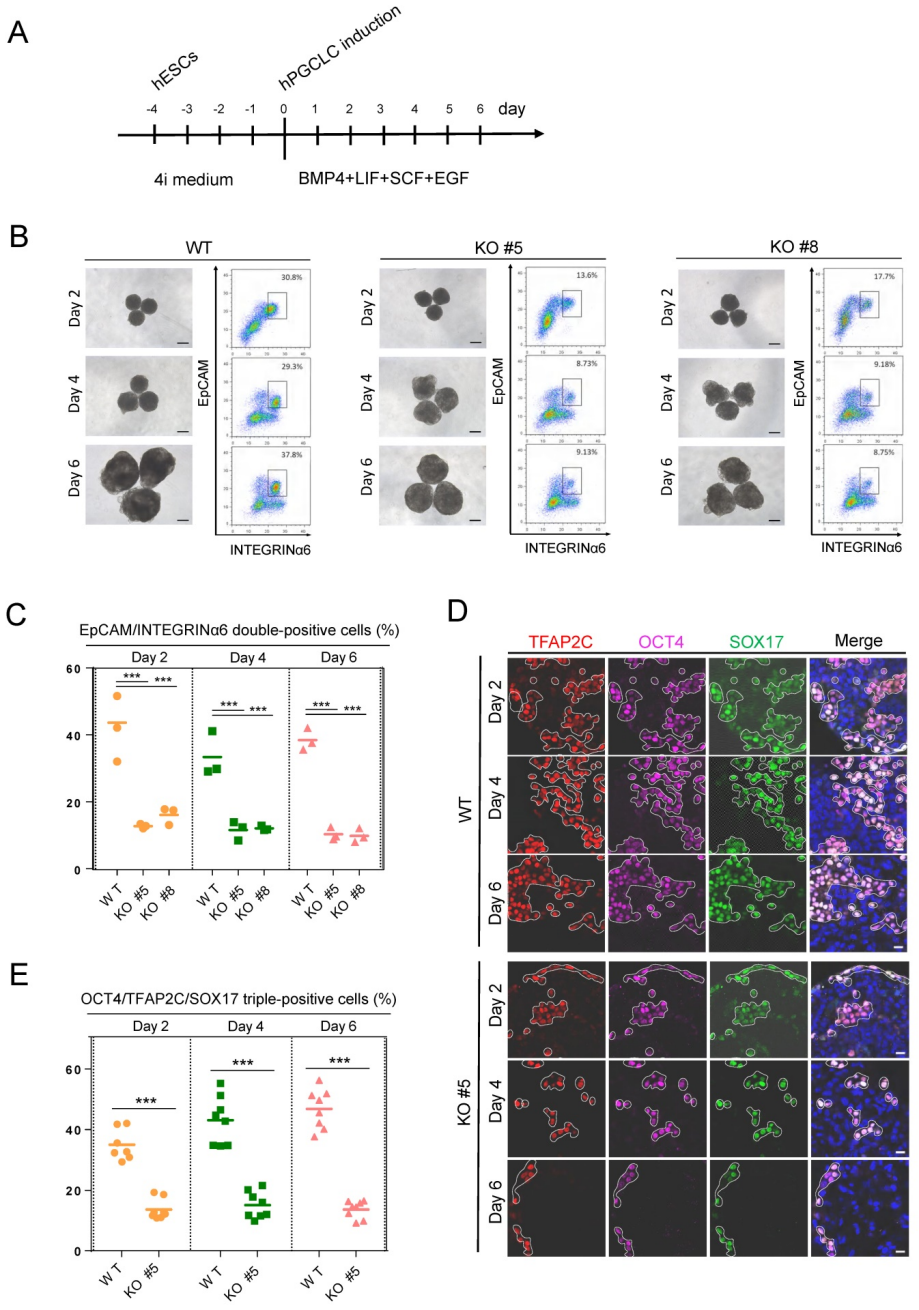
** KDM2B is required for hPGCLC specification.** (A) Schematic protocol for hPGCLCs specification from hESCs. (B) Bright-field (left) and FACS analyses for EpCAM/INTEGRINa6 expression (right) of floating aggregates of the WT and *KDM2B* KO #5, #8 hESCs upon hPGCLC induction at the indicated days. Percentages for EpCAM/INTEGRINa6 double-positive cells (rectangular gates) are shown. Scale bars, 250 µm. (C) Cell numbers per percentage of EpCAM/INTEGRINα6 double-positive cells for the WT and KO line; n = 3 in (B). Mean values are shown as bars. ***p < 0.0001. (D) Immunofluorescence analysis of OCT4, TFAP2C and SOX17 expression in day 2, 4 and 6 hPGCLCs from WT and *KDM2B* KO #5 hESCs. Scale bars, 20 µm. (E) Percentage of OCT4, TFAP2C and SOX17 triple-positive cells in day2, 4 and 6 hPGCLCs for WT and *KDM2B* KO #5 hESCs; n = 8 in (D). Mean values are shown as bars. ***p < 0.0001.

As KDM2B is predominantly expressed in human germ cells, we then explored the role of KDM2B in germ cell development. The *KDM2B* KO and WT control hESCs were cultured in a cocktail of inhibitors with four kinases (4i medium) for 4 days, then cells were incubated with hPGCLCs induction system including BMP4, stem cell factor (SCF), epidermal growth factor (EGF), and leukemia inhibitory factor (LIF) under a floating-aggregate condition for 6 days (Figure 2A). A time-dependent FACS analysis revealed that the hPGCLCs derived from *KDM2B* KO hESCs was significantly decreased as compared to WT controls (Figure 2B, C); furthermore, the expression of hPGCLC specification markers such as *SOX17, OCT4,* and *TFAP2C* in *KDM2B* KO hPGCLCs significantly reduced throughout the hPGCLC's development (Figure 2D, E), suggesting that KDM2B is involved in hPGCLC specification originated from hESCs.

### KDM2B orchestrates the early transcriptome transition and facilitates hPGCLC differentiation

**Figure 3:**
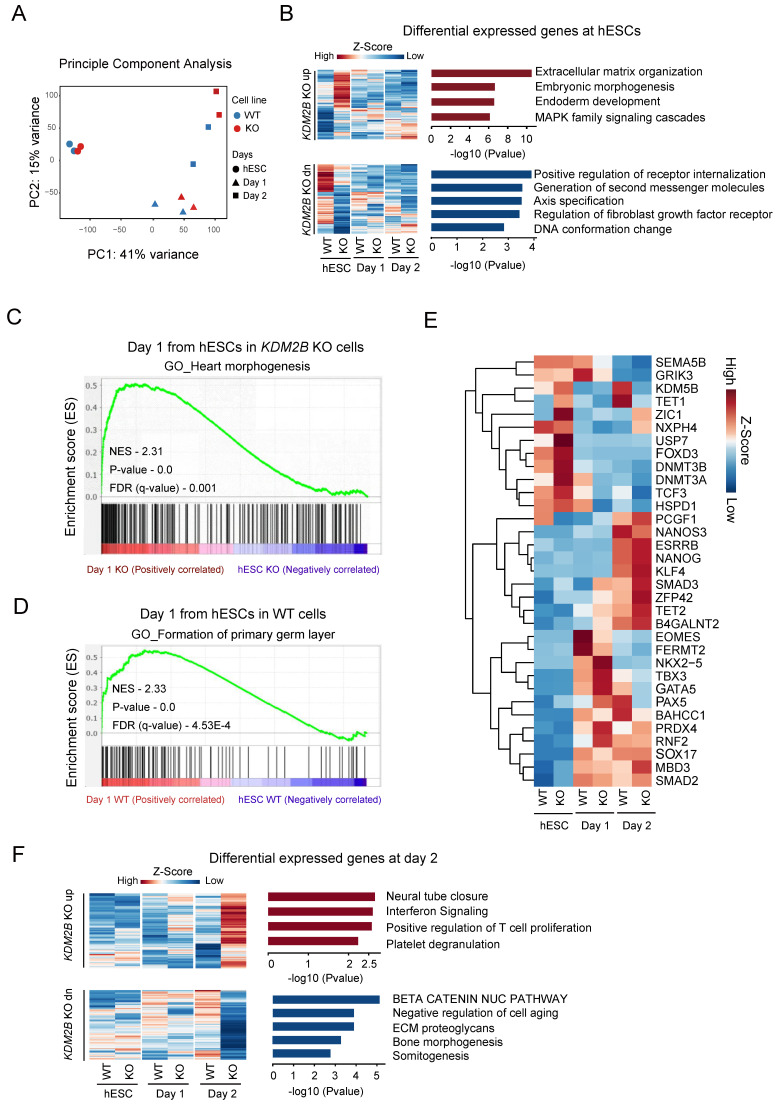
** Genes regulated by KDM2B for hPGCLC specification.** (A) Principle component analysis of RNA-Seq gene counts. Biological replicates are highlighted. (B) Gene Ontology analysis of upregulated (up) or downregulated (dn) genes in *KDM2B* KO hESCs. Genes above Padj <0.05 Fold change >1 cut-off was used to select the differentially expressed genes and shown as heatmap. Mean of two independent biological replicates is shown. (C, D) GSEA for differentiating day 1 cells from hESCs. The enrichment of gene sets was compared between KO versus WT. qvalue <0.05 cutoff was used to the select the gene network ontology. The P-values and NES (Normalized Enrichment score) are denoted in each plot. (E) Heatmap of gene expression (z-score) of key PGC-associated genes and pluripotency, self-renewal, endoderm, early development, DNA methylase and DNA demethylase markers. (F) Gene Ontology analysis of upregulated (up) or downregulated (dn) genes in response to *KDM2B* KO in day 2 hPGCLCs. Gene above Padj <0.05 Fold change >1 cut-off was used to select the differentially expressed genes and shown as heatmap. Mean of two independent biological replicates is shown.

To investigate the specific role of KDM2B in a stage specific manner, we performed a time dependent RNA-Seq on hESC, day 1 and day 2 with 2 biological replicates during hPGCLC differentiation process. The principle component analysis highlighted the transcriptional changes between WT and *KDM2B* KO cells started from day 1 onwards (Figure 3A). However, the differential expression (DE) analysis (gene cutoff: Padj < 0.05) revealed that genes annotated to somatic differentiation were up-regulated in the *KDM2B* KO cells, whereas genes regulated small molecules related receptor signaling, axis specification and DNA conformational changes were down-regulated in hESC stage (Figure 3B). To further explore the function of KDM2B in cell state transition from ESC to hPGCLC at early stage, we performed GSEA 31 with WT and *KDM2B* KO hPGCLCs after 1 day of induction. Intriguingly, we found that genes related to germ layer formation were more enriched in WT hPGCLC whereas genes associated with heart morphogenesis were enriched in *KDM2B* KO hPGCLCs (Figure 3C, D).

We next examined the expression of key pluripotency genes and epigenetic regulators specifically (Figure 3E). Most of pluripotency genes exhibited no significant differences between WT and *KDM2B KO* cells, however, some epigenetic regulators such as KDM5B, TET1 were significantly up-regulated after hPGCLC induction (from day 2) in KDM2B competent cells, while the activation of these genes' transcription were not observed in *KDM2B* KO hPGCLCs. Interestingly, we also observed inhibition of a key hPGCLC early cell fate transitional marker EOMES in *KDM2B* KO cells on day 1, which might explain the impaired capacity of *KDM2B* KO hPGCLCs formation. The Gene ontology analysis revealed those genes associated with neural tube closure and immunological response were up-regulated, but β-catenin pathway, bone morphogenesis and somitogenesis were inhibited in *KDM2B* KO hPGCLCs (Figure 3F). These data suggested that *KDM2B* deletion could cause some epigenetic factors alternation which subsequently affected cell fate determination during hPGCLC development.

### Induced expression of KDM2B re-establishes hPGCLCs specification

**Figure 4:**
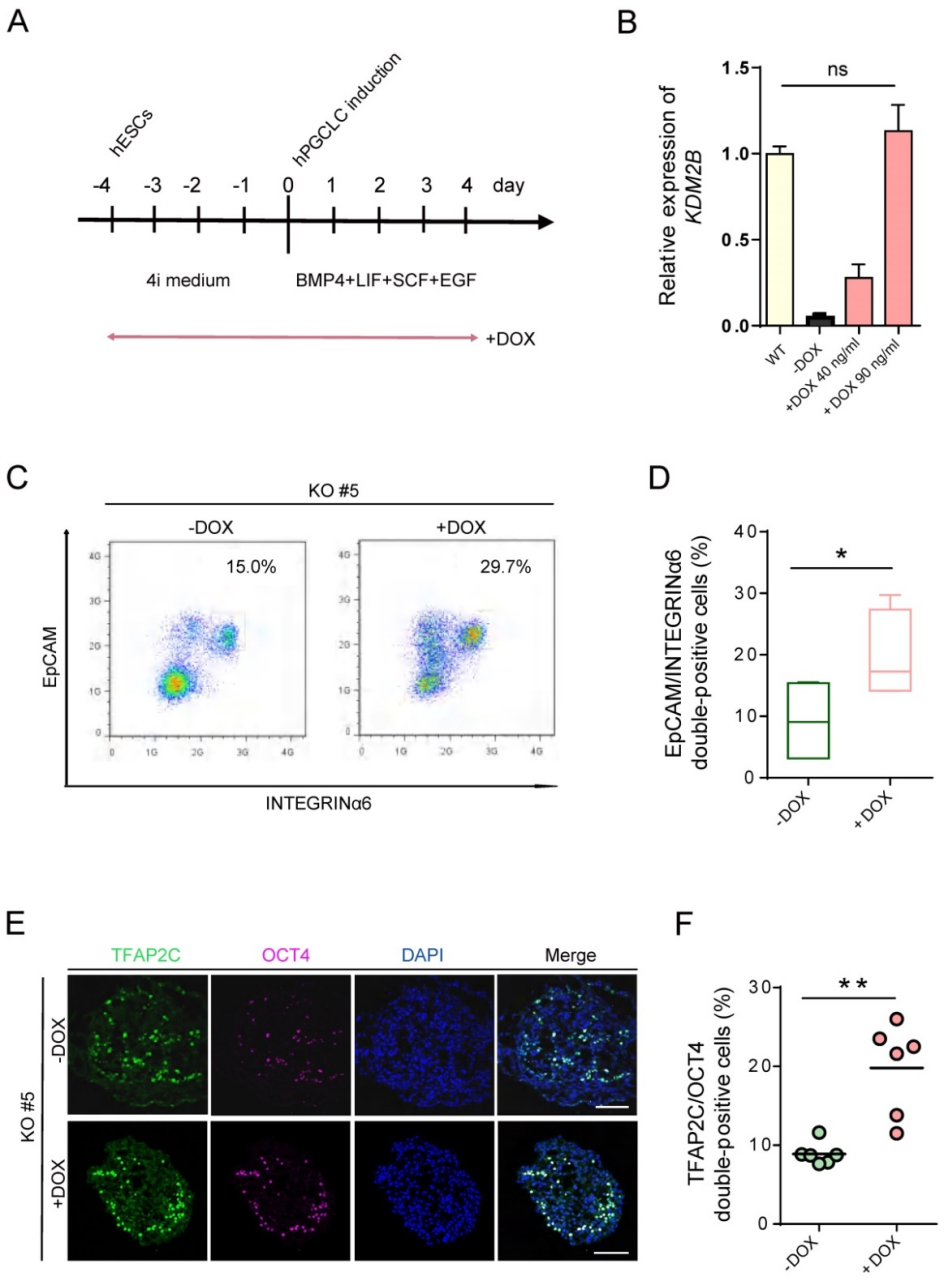
** hPGCLC specification defect can be rescued by induced expression of KDM2B.** (A) Scheme for the Tet-On inducible expression experiments. The timing of Dox administration is shown. (B) Relative mRNA levels of *KDM2B* in WT hESCs and induced expression of *KDM2B* KO #5 without (-Dox) or with 40 ng/mL and 90 ng/mL DOX, respectively (+Dox 40 ng/mL, +Dox 90 ng/mL). The level of KDM2B in WT hESCs is set as 1. Error bars indicate mean ± SD from three independent biological replicates. Ns refer to non-significant. (C) FACS analyses for EpCAM/INTEGRINα6 expression in day 4 hPGCLCs for *KDM2B* KO #5 without (-Dox) or with continuous administration of 90 ng/mL DOX (+Dox) throughout 4i hESC to hPGCLC development as in (A). Percentages for EpCAM/INTEGRINa6 double-positive cells (rectangular gates) cells are shown. (D) Percentage of EpCAM/INTEGRINα6 double-positive cells for *KDM2B* KO #5 without (-Dox) or with DOX administration (+DOX); n=4 in (C). Mean values are shown as bars. Error bars indicate mean ± SD. *p < 0.05. (E) Immunofluorescence analysis of TFAP2C and OCT4 in day 4 hPGCLCs for *KDM2B* KO #5 without (-Dox) or with continuous administration of 90 ng/mL DOX (+Dox) throughout 4i hESC to hPGCLC development as in (A). Scale bars, 100 µm. (F) Percentage of OCT4 and TFAP2C double-positive cells for *KDM2B* KO #5 without (-Dox) or with DOX administration (+DOX); n = 6 in (E). Mean values are shown as bars. **p < 0.005.

To determine whether re-expression of KDM2B could functionally rescue the deficiency of hPGCLC specification, we induced* KDM2B* expression with *KDM2B* KO #5 under the control of the Tet-on system, which would allow doxycycline (DOX) to activate KDM2B by duration and dosage (Figure 4A). Remarkably, 90 ng/mL DOX was sufficient to activate the expression of *KDM2B* to the endogenous level (Figure 4B). More importantly, continuous induction of the *KDM2B* expression throughout 4i hESC to hPGCLC development significantly increased the EpCAM/INTEGRINα6 double-positive hPGCLC production as compared to non-induced *KDM2B* KO hESCs at day 4 (Figure 4C, D). Accordingly, immunofluorescence analyses revealed the restoration of TFAP2C and OCT4 expression (Figure 4E, F). Since the *KDM2B* KO phenotype can be functionally rescued by KDM2B re-expression, we thus demonstrate the hPGCLC specification defect result from the loss of *KDM2B*, but not from off-target effects.

## Discussion

It is estimated that infertility affects 10-15% of people of reproductive age worldwide, and male infertility contributes half of all infertility cases 32. The targeted differentiation of iPSCs into germ cells from infertility males allows patients to have their own sperms, however, the* in vitro* cultured hPGCLCs do not progress beyond the pre-migratory stage and thus do not undergo fully epigenetic reprogramming or activation of meiotic genes 33, 34. The understanding of hPGC/ hPGCLCs specification achieved great progress in recent years particularly the discovery of a distinct regulatory network for specification. For instance, SOX17 is reported to be a key regulator of hPGCLC specification, which acts as the upstream regulator for genes such as *BLIMP1* to initiate the human germ cell transcription 8. Furthermore, studies in the iMeLC differentiation system revealed that *EOMES* was first activated by WNT signaling, which in turn regulated *SOX17* expression in hPGCLCs 9, and this specific regulatory network was different from mouse germ cell specification.

Recently, accumulating evidence demonstrated that methylation of transcription start sites (TSSs) of whole genome was maintained at low levels during mouse and human PGCLC specifications 7, 35. The chromatin repression state is altered during the migration of PGCs, initiating the genome reprogramming of PGCs, which is essential for the postzygote to acquisition of totipotency 36, 37. For example, Dnmt3a mutant mice showed impaired spermatogenesis with loss of two third DNA methylation modifications of the paternal imprinting loci in their spermatogonia 38; another study observed abnormal alterations in DNA methylation patterns at paternally imprinted sites in the sperm collected from patients with severe oligozoospermia 39. Moreover, histone demethylase with catalytic methylation modifications was identified as an important epigenetic regulator of spermatogenesis and required for proper spermatogenesis 40. Deletion of H3K4 demethylase Kdm5b/Jarid1b affected the fertility of female mice 41. Although knocking out of H3K36me1/2 demethylase (which inhibited the initiation of cryptic transcription) Kdm2a/Jhdm1a had no effect on the maintenance of pluripotency in mESCs, but it directly impaired germ cell gene expression in mouse primordial germ cell-like cells 42. However, the detailed mechanisms of how histone methylation modifications regulate male germ cell processes require further investigation.

In our study, we first observed particularly high expression of the histone demethylase KDM2B in male FGCs but not in male somatic cells. Paradoxically, unlike previously reported mice data that knockdown of Kdm2b significantly affected mESC pluripotency 20, *KDM2B* deficient hESCs could still express pluripotency genes, such as OCT4, NANOG, as well as SOX2, and transplantation into immuno-deficient mice can form teratomas. Therefore, our data further support that deletion of *KDM2B* had no significant effect on pluripotency in hESC. Importantly, we also demonstrated that depletion of KDM2B dramatically impaired hPGCLCs' differentiation whereas ectopically expressed *KDM2B* could efficiently rescue such defect. Mechanistically, as revealed by the transcriptional profiling, knockout of *KDM2B* caused up-regulation of embryonic morphogenesis and extracellular matrix organization whereas inhibition of cell signal transduction and DNA conformation associated genes' expression. We also found that during hPGCLC differentiation, germline genes were more enriched in WT hPGCLC but heart morphogenesis as well as endodermal or ectodermal genes was up-regulated in *KDM2B* KO hPGCLC, suggesting the *KDM2B* KO cells were prone to undergo somatic differentiation. Hence, KDM2B might play a role in suppressing somatic gene expression and transformation towards somatic cells during the specification of PGCs.

The RNA-Seq data also revealed that both pluripotency-related genes (e.g. *ESRRB, NANOG, KLF4, ZFP42, and MBD3*) and self-renew genes (e.g. *SMAD2, SMAD3)* were up-regulated in *KDM2B* KO hESCs 2 days after hPGCLC induction, indicating the inability of such cells to completely escape pluripotency state. Furthermore, we found that germ cell associated genes (e.g. *SOX17, EOMES* and *NANOS3*) were down-regulated in *KDM2B* KO hPGCLCs, which was consistent with the impaired specification, observed in *KDM2B* KO derived cells. Finally, many other DNA methylation regulators, including *DNMT3A, DNMT3B* and *TET1* were significantly changed upon *KDM2B* deletion, suggesting the absence of KDM2B may affect epigenetic profile during hPGCLC specification and dys-regulated DNA methylation might lead to transcriptional variation of target genes. Since the global H3K4me3 and H3K36me2 levels were upregulated in *KDM2B* KO hESCs, we therefore propose that KDM2B regulates hPGCLC's specification may depend on its demethylase activity. Taken together, our study indicates that KDM2B is an indispensable epigenetic regulator for hPGCLC specification.

## Supplementary Material

Supplementary figures and tables.

Supplementary document - differentially expressed gene list.
