# Supplementary material for: The histone demethylase KDM2B regulates human primordial germ cell-like cells specification
Source: Int J Biol Sci. 2021 Jan 1;17(2):527–38. doi: 10.7150/ijbs.55873 (PMC7893587; doi:10.7150/ijbs.55873)
Supplement: Supplementary file 1 — Supplementary figures and tables. [file ijbsv17p0527s1.pdf]

Figure S1. Establishment of *KDM2B* Knockout hESC Lines with CRISPR/ Cas9 system.

(A-B) Targeting strategy of *KDM2B* knockout in hESC with the designated guide RNA (gRNA) and the resulting deleted sequences.

(C) Schematic representation of different truncated KDM2B mutant proteins.

Figure S2. *KDM2B* KO hESCs maintain pluripotent characteristics.

(A) Immunofluorescence analysis of pluripotency markers in WT and *KDM2B* KO #5 hESCs. Scale bars, 20 μm. In all panels, One representative experiment is shown out of the three replicate experiments.

(B) Relative mRNA levels of pluripotency marker genes in WT and *KDM2B* KO #5, #8 and #18 hESCs. Error bars indicate mean ± SD from three independent biological replicates. Ns refer to non-significant.

(C) HE staining of the teratoma sections derived from WT or *KDM2B* KO #5 hESCs. The teratoma tissues contained glandular epithelium (g, endoderm), cartilage (c, mesoderm) and neural epithelium (n.e., ectoderm). Scale bars, 20 μm.

A

Table of efficiency

	Sequenced	KO	Others WT or 3n frameshift	Heterozygous
KDM2B	17	7 (41%)	8 (47%)	2 (11.8%)

B

CRISPR targets and resulting frameshift mutation

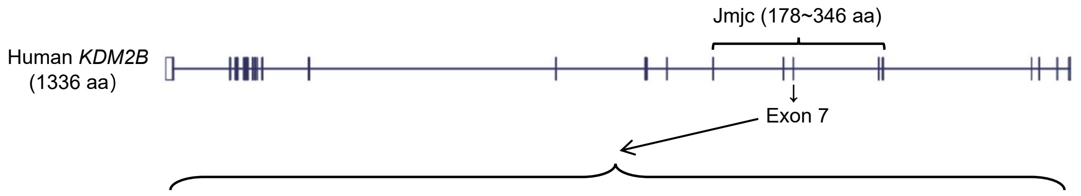

WT 5' GACTTCCACATCGACTTTGGAGGCACTTCCGTTTGGTACCATGTTTTCCGGGGTGGGAAG 3'

KDM2B KO #5 5' GACTTCCACATCGACTTTGGAGGCACTTCCCGTTTGGTACCATGTTTTCCGGGGTGGGAAG 3' 1 bp ins
KDM2B KO #8 5' GACTTCCACATCGACTTTGGAGGCAC---------GTTTGGTACCATGTTTTCCGGGGTGGGAAG 3' 4 bp del
5' GACTTCCACATCGACTTTGGAGGCACTTCC--------------------ATGTTTTCCGGGGTGGGAAG 3' 10 bp del
KDM2B KO #18 5' GACTTCCACATCGACTTTGGAGGCACTTCCCGTTTGGTACCATGTTTTCCGGGGTGGGAAG 3' 1 bp ins
5' GACTTCCACATCGACTTTGGAGGCA------------------------------TGTTTTCCGGGGTGGGAAG 3' 16 bp del

C

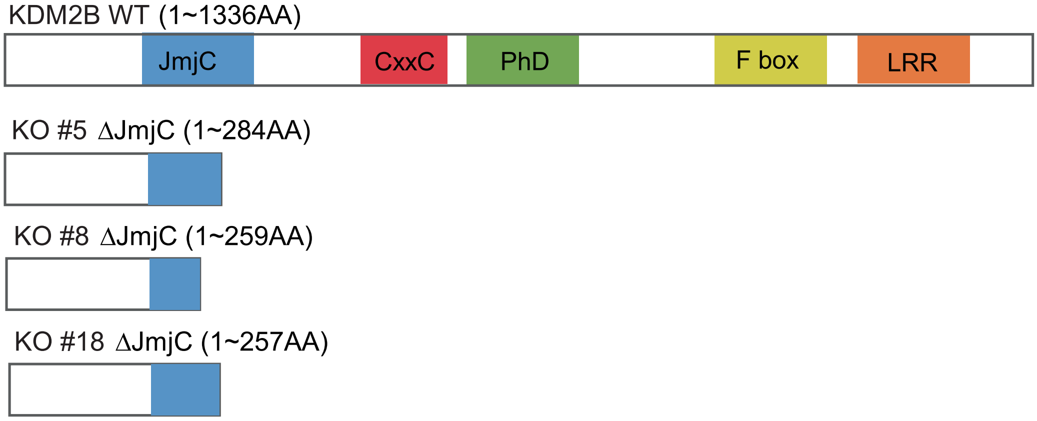

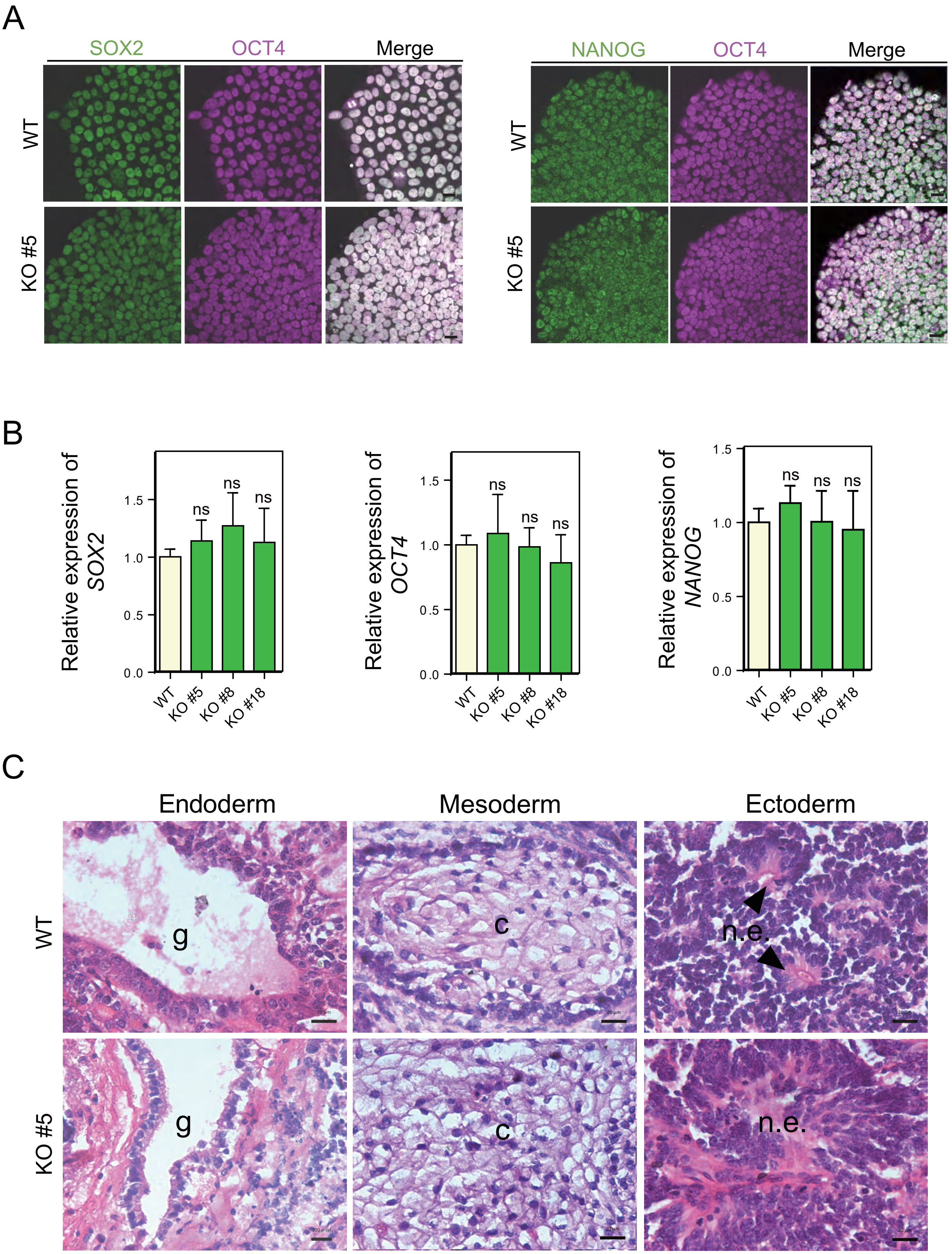
A
SOX2
OCT4
Merge
NANOG
OCT4
Merge
WT
KO #5
B
Relative expression of SOX2
Relative expression of OCT4
Relative expression of NANOG
0.0
0.5
1.0
1.5
ns
WT
KO #5
KO #8
KO #18
C
Endoderm
Mesoderm
Ectoderm
g
c
n.e.

Table S1. Primers for CRISPR/ Cas9.

TARGET		5’→ 3’
hKDM2B	Forward	CACCGACTTTGGAGGCACTTCCGTT
hKDM2B	Reverse	AAACAACGGAAGTGCCTCCAAAGTC

Table S2. List of primers for qPCR.

TARGET		5’→ 3’
hGAPDH	Forward	CGCTTCGCTCTCTGCTCCTCCTGT
hGAPDH	Reverse	GGTGACCAGGCGCCCAATACGA
hACTIN	Forward	ATAGCAACGTACATGGCTGG
hACTIN	Reverse	CACCTTCTACAATGAGCTGC
hARBP	Forward	GAAACTCTGCATTCTCGCTTCC
hARBP	Reverse	ACTCGTTTGTACCCGTTGATGA
hSOX2	Forward	ACACCAATCCCATCCACACT
hSOX2	Reverse	CCTCCCCAGGTTTTCTCTGT
hNANOG	Forward	TGCTGAGATGCCTCACACGGA
hNANOG	Reverse	TGACCGGGACCTTGTCTTCCTT
hOCT4	Forward	GCTGGAGCAAAACCCGGAGG
hOCT4	Reverse	TCGGCCTGTGTATATCCCAGGGTG
hKDM2B	Forward	TTCCGGGGTGGGAAGATTT
hKDM2B	Reverse	GGCATGGATCCAACCGGAA

Table S3. List of antibodies used in the study.

REAGENT or RESOURCE	SOURCE	IDENTIFIER
Rabbit anti-Sox2	Abcam	Cat#Ab97959
Goat anti-Nanog	R&D Systems	Cat#AF1997
Goat anti-Oct4	Santa Cruz, Dallas	Cat#sc-5279
Goat anti-Sox17	R&D Systems	Cat#AF1924
Rabbit anti-TFAP2C	Santa Cruz, Dallas	Cat# sc-8977
Rabbit anti-β-Actin	Proteintech	Cat#20536-1-AP
Rabbit anti-H3K4me3	Millipore	Cat#07-473
Rabbit anti-H3K36me2	Abcam	Cat# ab9049
Rabbit anti-Flag	Sigma	Cat# F1804
Rabbit anti-KDM2B	Merck Millipore	Cat#09-864
Goat Alexa Fluor 647 anti-mouse IgG	Jackson ImmunoResearch	Cat #115-545-003
Goat Alexa Fluor 594 anti-rabbit IgG	Jackson ImmunoResearch	Cat #111-585-003
Goat Alexa Fluor 488 anti-mouse IgG	Jackson ImmunoResearch	Cat #115-545-003
Donkey Cy2 anti-goat IgG	Jackson ImmunoResearch	Cat #705-225-147
Anti-rabbit HRP	ZSJB-BIO	Cat #zb2301
Anti-mouse HRP	ZSJB-BIO	Cat #zb2305
